# Reliability of foveal avascular zone measurements in eyes with retinal vein occlusion using optical coherence tomography angiography

**DOI:** 10.1186/s40942-020-00237-w

**Published:** 2020-08-03

**Authors:** Bruno Mauricio Rodrigues de Oliveira, Luis Filipe Nakayama, Bruno Rebello de Godoy, Alexandre Gomes Bortoloti de Azevedo, Flavio Eduardo Hirai, Somaia Mitne

**Affiliations:** Retina and Vitreous Sector, Department of Ophthalmology, Universidade Federal de São Paulo – Escola Paulista de Medicina, Rua Botucatu, 821, Vila Clementino, São Paulo, SP 04023-062 Brazil

**Keywords:** Retina, Foveal avascular zone, OCT, Retinal vein occlusion

## Abstract

**Background:**

To evaluate the reliability of foveal avascular zone (FAZ) area measurements using optical coherence tomography angiography (OCTA) in eyes with retinal vein occlusion (RVO).

**Methods:**

Twenty-five OCTA exams of patients with RVO were evaluated retrospectively. Three examiners performed manual measurements of the FAZ, and interrater and intrarater reliability were obtained.

**Results:**

The intraclass correlation coefficient (ICC) for interrater reliability for individual measurements was 0.62 (moderate) with a 95% confidence interval (CI) of 0.40 to 0.79 (p < 0.001). The ICC (95% CI) for intrarater reliability was 0.92 (0.82 to 0.96) for rater A, 0.96 (0.91 to 0.98) for B, and 0.88 (0.76 to 0.94) for C (p < 0.001). In all subanalyses including presence of edema and type of occlusion, interrater reliability was poor/moderate, and intrarater reliability was good/excellent.

**Conclusion:**

The FAZ varies significantly among eyes with RVO, so measurements obtained using OCTA should be analyzed with caution due to the moderate level of reliability among different examiners.

## Background

Retinal vein occlusion (RVO) is a common retinal vascular disease that may lead to significant visual morbidity. The vascular obstruction can occur either in the central retinal vein or in a branch of the retinal vein [1–3]. Systemic vascular diseases are the most important risk factor for RVO, especially in elderly patients. Hypercoagulable states and systemic inflammatory conditions are also risk factors that must be considered in young patients, in whom RVO is uncommon.

Visual outcome depends on the severity of retinal ischemia and macular edema. Hence, evaluation of the retinal vasculature is important for determining the therapeutic strategy and prognosis [1–3].

Fluorescein angiography (FA) has traditionally been used to analyze retinal capillary non-perfusion and neovascularization. FA is an invasive procedure involving intravenous dye injection that provides two-dimensional images with dynamic visualization of blood flow. Therefore, patterns of leakage, pooling and staining can be observed and correlated with clinical presentation. However, FA is unable to analyze the different retinal vasculature layers separately [4, 5].

The optical coherence tomography angiography (OCTA) allows visualization of vascular maps of the retina and choroid separated by layers and non-invasively [6, 7]. OCTA can provide structural and functional information on the retina and choroidal vascularization and detect vascular flow at a fixed point in time [6]. In addition, due to the high resolution of the capillary information, OCTA permits measurement of the dimensions of the foveal avascular zone (FAZ). Previous studies have demonstrated the reliability of FAZ measurements by OCTA compared with the contralateral eye, albeit with great interobserver variability [8–10]. However, software platforms lack normalized data to determine whether microvascular changes are abnormal, and the reliability of FAZ measurements using OCTA in vascular retinal diseases is unclear [11, 12].

The aim of this study was to evaluate the reliability of FAZ area measurements in eyes with RVO obtained by swept-source OCTA using a Topcon DRI OCT Triton.

## Methods

OCTA exams of consecutive patients attending retinal clinics at Federal University of São Paulo and diagnosed with RVO in the last 2 years were analyzed retrospectively. This study was approved by the UNIFESP Institutional Review Board and carried out in accordance with the tenets of the Declaration of Helsinki (Additional file 1).

The inclusion criteria were diagnosis of RVO (central or branch of the retinal vein) according to clinical evaluation and fundus retinal exam performed by at least two retina specialists. Patients were excluded from this study if their medical records documented a history of another ocular or clinical disease that may lead to retinal vascular abnormalities.

A Topcon DRI Triton swept-source optical coherence tomographer (Topcon Corporation, Japan) was used to obtain volumetric angiography maps of the retina. All exams met the quality thresholds given by the OCT software with a minimum index of 40. A macular scan size of 6 × 6 mm was used for FAZ evaluation at the superficial retinal vascular layer. Manual measurements were performed using the built-in IMAGEnet^®^ software (Topcon Medical Systems, Inc.). Two retina specialists and one-third-year ophthalmology resident were recruited to perform the measurements of all patients blinded to each other’s results.

Every examiner performed two measurements of each image at different times with an interval of at least 3 weeks between the two measurements. Before measurement, evaluation and correction of the segmentation of retinal layers for angiography analysis were performed at the discretion of the examiner. A previous study reported the importance of adjusting segmentation, including the full thickness of the retina, in order to reduce errors and variations [13].

Statistical analysis was performed with Stata v.14 (College Station, Texas, USA). To evaluate the interrater (*between*-*observer*) reliability of the measurements, we calculated the intraclass correlation coefficient (ICC) using a two-way random-effects model evaluating single raters for absolute agreement. For the intrarater (*within*-*observer*) reliability test, we used a two-way mixed-effects model for absolute agreement between measurements within the rater.

The ICC takes a value from zero (no agreement) to one (complete agreement). For analysis purposes, we classified ICC values as follows: 0 to 0.50, poor reliability; 0.50 to 75, moderate reliability; 0.75 to 0.90, good reliability; and greater than 0.90, excellent reliability.

## Results

Twenty-five patients were enrolled in this study, including 14 (56%) males and 11 (44%) females. The diagnosis was central RVO in 14 patients (56%) and branch RVO in 11 patients (44%). Eighteen patients (72%) presented OCT scans with macular edema. The mean age of the patients was 56.7 (SD 12.5) years, with a range from 30 to 78 years (Table 1).

**Table 1:** Baseline characteristics of patients

Characteristics	Sample (total = 25)
Gender	
Male	14 (56%)
Female	11 (44%)
Type of occlusion	
Central vein	14 (56%)
Branch vein	11 (44%)
Mean age (SD)	56.7 (12.5)
Presence of macular edema	18 (72%)
Affected eye	
Right eye	11 (44%)
Left eye	14 (56%)

The individual FAZ measurements of the patients are shown in Table 2, along with the mean FAZ dimensions provided by each examiner (A, B and C). To evaluate the consistency of the measurement process, statistical analysis was performed using the ICC to determine the repeatability (intrarater reliability) and reproducibility (interrater reliability).

**Table 2:** Foveal avascular zone (FAZ) measurements of each examiner

	FAZ A1	FAZ A2	Mean A	FAZ B1	FAZ B2	Mean B	FAZ C1	FAZ C2	Mean C
1	529.453	519.609	524.531	986.836	1094.766	1040.801	862.646	835.313	848.9795
2	312.539	361.758	337.1485	355.078	285.5	320.289	359.297	451.406	405.3515
3	665.859	848.32	757.0895	351.563	375.117	363.34	316.758	412.734	364.746
4	529.805	502.031	515.918	531.211	525.586	528.3985	567.07	576.211	571.6405
5	880.312	582.188	731.25	504.492	547.031	525.7615	519.258	512.578	515.918
6	545.625	926.367	735.996	411.328	525.234	468.281	672.891	1050.82	861.8555
7	959.766	941.484	950.625	724.219	687.305	705.762	685.195	680.625	682.91
8	392.695	273.516	333.1055	1174.219	1086.68	1130.4495	1406.602	1087.734	1247.168
9	627.188	387.422	507.305	423.984	421.523	422.7535	413.086	387.07	400.078
10	338.203	289.336	313.7695	443.32	368.789	406.0545	365.625	358.242	361.9335
11	696.797	685.195	690.996	421.875	837.773	629.824	1147.5	770.273	958.8865
12	413.086	533.32	473.203	354.375	348.398	351.3865	288.281	241.172	264.7265
13	1506.797	1195.313	1351.055	773.438	1213.594	993.516	929.531	1499.063	1214.297
14	1376.719	1406.953	1391.836	1429.102	1595.742	1512.422	1495.47	1514.18	1504.825
15	206.719	265.43	236.0745	63.281	72.422	67.8515	80.156	85.43	82.793
16	844.102	731.602	787.852	684.492	688.008	686.25	622.969	427.5	525.2345
17	566.367	492.188	529.2775	601.172	759.375	680.2735	390.586	385.684	388.135
18	1523.672	1567.07	1545.371	1978.945	2036.602	2007.7735	366.328	309.727	338.0275
19	661.641	648.984	655.3125	224.648	208.125	216.3865	184.922	145.625	165.2735
20	706.992	736.523	721.7575	355.586	364.57	360.078	342.773	248.203	295.488
21	145.195	192.656	168.9255	76.641	74.883	75.762	218.32	235	226.66
22	265.078	315	290.039	229.57	239.06	234.315	140.977	146.25	143.6135
23	356.836	359.648	358.242	318.516	312.188	315.352	253.447	262.969	258.208
24	368.086	458.086	413.086	485.156	464.063	474.6095	317.461	348.398	332.9295
25	418.359	557.227	487.793	75.937	93.164	84.5505	396.211	354.375	375.293

The ICC for interrater reliability for individual measurements was 0.62 (moderate), with a 95% confidence interval (CI) of 0.40 to 0.79 (p < 0.001). When considering all raters as a group and analyzing the reliability between the average measurements, the ICC value was 0.83 (good), with a 95% CI of 0.67 to 0.92 (p < 0.001).

The ICC (95% CI) for intrarater reliability was 0.92 (0.82 to 0.96) for rater A, 0.96 (0.91 to 0.98) for rater B, and 0.88 (0.76 to 0.94) for rater C; these differences were significant (p < 0.001).

Considering macular edema and type of occlusion (central or branch RVO), the ICC for interrater reliability for individual measurements was 0.75 (moderate) for central RVO (95% CI 0.51–0.90 and p < 0.001); 0.48 (poor) for branch RVO (95% CI 0.12–0.80 and p < 0.05); 0.62 (moderate) for macular edema (95% CI 0.36–0.82 and p < 0.001); and 0.58 (moderate) for the group without macular edema (95% CI 0.11–0.90 and p < 0.05).

For the above subanalyses, the intrarater reliabilities of raters A, B and C were all good/excellent with statistical significance (p < 0.001), as shown in Table 3. There was no difference in reliability comparing different types of occlusion or presence of edema in the intrarater analysis.

**Table 3:** Intraclass correlation coefficient (ICC) for reliability analysis of foveal avascular zone (FAZ) measurements

Rating	ICC	95% conf. interval	
Interrater reliability			
Individual	0.62	0.41	0.79
Average	0.83	0.67	0.92
Intrarater reliability			
A	0.92	0.82	0.96
B	0.96	0.91	0.98
C	0.88	0.76	0.94
Interrater reliability—type of occlusion			
Central vein occlusion*			
Individual	0.75	0.51	0.90
Average	0.90	0.76	0.96
Branch vein occlusion**			
Individual	0.48	0.12	0.80
Average	0.74	0.29	0.92
Interrater reliability—presence of edema			
Macular edema			
Individual	0.62	0.36	0.82
Average	0.83	0.63	0.93
Without macular edema			
Individual	0.58	0.11	0.90
Average	0.81	0.28	0.96
Intrarater reliability—type of occlusion			
Central vein occlusion			
A	0.91	0.75	0.97
B	0.99	0.96	0.99
C	0.94	0.81	0.98
Branch vein occlusion			
A	0.93	0.75	0.98
B	0.94	0.80	0.98
C	0.79	0.40	0.94
Intrarater reliability—presence of edema			
Macular edema			
A	0.91	0.78	0.97
B	0.93	0.83	0.97
C	0.87	0.69	0.95
Without macular edema			
A	0.91	0.57	0.98
B	0.98	0.87	0.99
C	0.90	0.53	0.98

p < 0.001, *p < 0.001, **p < 0.05

## Discussion

The FAZ is the macular capillary-free zone surrounded by interconnected capillary vessels. Its size correlates with the foveal circulation condition in retino-vascular diseases [1–3]. Previous studies have suggested a mean physiological FAZ area of 200 to 400 μm^2^ in healthy subjects [8]. RVO leads to FAZ enlargement, and measurements of the FAZ therefore provide an objective evaluation of macular ischemia and consequently visual acuity prognosis [1].

Fluorescein angiography (FA) is the standard exam for FAZ evaluation, but the high variability in measurements diminishes the reliability of this method, even in healthy patients [8]. FA may also miss some microvasculature changes that are more readily observed in OCTA, including deep capillary plexus, which is mainly affected in RVO [1, 11]. Moreover, the FA exam cannot be performed in pregnant women and patients with fluorescein allergy, renal failure, severe asthma or significant cardiac disease [5].

OCTA is a dye-less method of imaging retinal circulation in different layers that allows a volumetric approach.

To avoid segmentation artifact manual correction of automatic retinal layers segmentation were performed in all OCT exams*. En face* retinal exam were evaluated to identify hemorrhages or opacities that could lead to shadowing and projection artifacts. In every OCTA exam artifacts must be considered and manual correction should be performed to decrease artifacts in exam and *en face* exam need to be analyzed altogether with OCTA vascular exam to reduce shadowing and projections artifacts.

Previous OCTA studies in healthy patients have suggested excellent reproducibility and repeatability in measurements of the FAZ [8], but few studies have considered macular pathologies such as macular ischemia or edema [14]. Although several studies have aimed to correlate OCTA findings such as enlargement of FAZ area, vascular network attenuation and retinal nonperfusion with the severity of retinal vascular diseases [2, 15–19], the reasons for the large variability in prognosis among patients and the role and impact of such anatomic features in clinical outcomes remain unclear [2]. Previous reports have identified qualitative and quantitative changes associated with RVO via OCTA. However, whether the quantitative data provided by OCTA software are accurate and can be correlated with macular function have not been established [11].

**Fig. 1:**
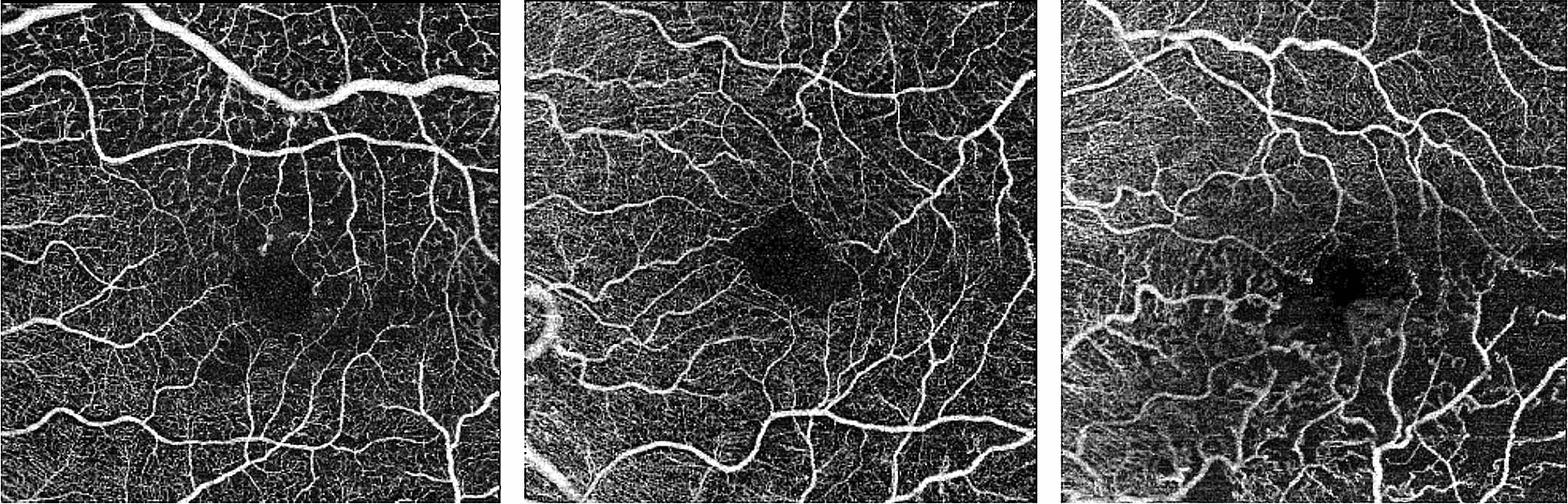
OCTA maps of three different subjects with great variability of foveal avascular zone

The present report indicated good/excellent intrarater reliability of manual FAZ measurements and satisfactory repeatability of FAZ area measurements via OCTA. By contrast, interrater reliability (i.e., reproducibility) was moderate, suggesting that FAZ measurements by different observers may not be comparable. Moreover, the great variability of FAZ dimensions (as shown in Table 2 and Fig. 1) make correlations with disease severity difficult. In addition, the present study did not demonstrate whether the type of occlusion and the presence of macular edema are factors that impact the reliability of FAZ measurements, even after review and correction of the segmentation of retinal layers for angiography analysis.

The main concern about OCTA image interpretation is the presence of artifacts, specially segmentation, projection, and masking artifacts. As mentioned above, segmentation artifacts can be reduced by using manual correction of retinal layers. Also, evaluation of *en face* retinal maps could avoid projection artifacts misinterpretation. However, a pronounced edema or highly reflective intraretinal structures could produce masking artifacts in underlying layers [20]. Previous reports noticed the absence of capillaries detection in the areas of retinal cysts [21, 22]. Couturier et al. hypothesized that retinal cysts provoke a displacement of the capillary in the cysts edges or more likely the cysts develop preferentially in nonperfusion areas [21]. Sellam et la reported that after cyst regression only 36% of the eyes improved vascular density in these areas [22].

The large number of artifacts in OCTA images segmentation and masking effects may complicate the proper judgement of FAZ limits, resulting in an irregular and inaccurate vascular map close to the fovea. Consequently, the results may not be interchangeable among patients. However, the good intrarater reliability observed in the present study suggests that the use of FAZ measurements for individual follow-up is feasible.

## Conclusion

In summary, caution is advised when analyzing measurements of the FAZ area in eyes with RVO. Comparisons between examiners provide only moderate reliability, and the results may not be interchangeable.

## Supplementary information

**Additional file 1.** Ethics Committee Approval provided by UNIFESP Institutional Review Board.

## Data Availability

The datasets generated during the current study that were used to calculate the primary outcome parameters are available upon reasonable request from the corresponding author Oliveira, BMR.
