# Supplementary material for: Reliability of foveal avascular zone measurements in eyes with retinal vein occlusion using optical coherence tomography angiography
Source: Int J Retina Vitreous. 2020 Aug 3;6:35. doi: 10.1186/s40942-020-00237-w (PMC7398327; doi:10.1186/s40942-020-00237-w)
Supplement: Supplementary file 1 — Additional file 1. Ethics Committee Approval provided by UNIFESP Institutional Review Board. [file 40942_2020_237_MOESM1_ESM.pdf]

PARECER CONSUBSTANCIADO DO CEP

DADOS DO PROJETO DE PESQUISA

Título da Pesquisa: Confiabilidade da medida da zona avascular foveal em olhos com oclusão venosa de retina usando angiografia por tomografia de coerência óptica

Pesquisador: Bruno Mauricio Rodrigues de Oliveira

Área Temática:

Versão: 2

CAAE: 12801919.3.0000.5505

Instituição Proponente: Universidade Federal de São Paulo

Patrocinador Principal: Financiamento Próprio

DADOS DO PARECER

Número do Parecer: 3.445.580

Apresentação do Projeto:

Projeto CEP/UNIFESP n: 0492/2019 (parecer final)PROJETO APROVADO

Trata-se de projeto de Especializacao de Bruno Mauricio Rodrigues de Oliveira.

Equipe: Bruno Rebello de Godoy; Alexandre Gomes Bortolotti de Azevedo.

Orientadores: Prof(a). Dr(a). Somaia Mitne; Flavio Hirai; Nilva Simeren Bueno de Moraes.

Projeto vinculado ao Departamento de Departamento de Oftalmologia e Ciencias Visuais, Campus Sao Paulo, Escola Paulista de Medicina, UNIFESP.

-As informacoes elencadas nos campos "Apresentacao do Projeto", "Objetivo da Pesquisa" e "Avaliacao dos Riscos e Beneficios" foram retiradas do arquivo Informacoes Basicas da Pesquisa (PB_INFORMACOES_BASICAS_DO_PROJETO_1329621.pdf, postado em 29/04/2019). APRESENTACAO: A oclusao venosa de retina e uma doenca comum capaz de causar importante morbidade. Tradicionalmente, o exame de escolha para avaliar perfusao vascular retiniana e a angiofluoresceinografia. Mais recentemente, o avanco da tecnologia da tomografia de coerencia optica permite que mapas angiograficos da retina sejam obtidos de maneira nao invasiva. Sua alta resolucao de imagem permite avaliar ate mesmo os capilares da retina, sendo possivel medir as dimensoes da zona avascular foveal. Contudo, nao se sabe qual a confiabilidade e variabilidade dessas medidas.

O objetivo do presente estudo e avaliar a confiabilidade das medidas da zona avascular foveal em

pacientes com oclusao venosa retiniana a partir do uso de tomografia de coerencia optica.

HIPOTESE: A mensuracao da zona avascular foveal a partir dos mapas angiograficos do exame de tomografia de coerencia optica podem ser confiaveis e reprodutiveis entre diferentes observadores.

Objetivo da Pesquisa:

Objetivo Primario: O objetivo deste estudo e o de avaliar a confiabilidade das medicoes da area da ZAF usando Topcon DRI OCT Triton Swept Source entre diferentes examinadores.

Avaliação dos Riscos e Benefícios:

Em relacao aos riscos e beneficios, o pesquisador(a) declara:

Riscos: Risco de exposicao de dados de exames pessoais.
Beneficios: Não há benefícios diretos envolvidos na participação do presente estudo, contudo há benefícios indiretos de contribuição para melhor entendimento das alterações vasculares apresentadas em oclusões venosas retinianas em exame de tomografia de coerência óptica.

Comentários e Considerações sobre a Pesquisa:

TIPO DE ESTUDO: observacional retrospectivo.
LOCAL: ambulatorio de Retina e Vitreo do Departamento de Oftalmologia e Ciencias Visuais da Universidade Federal de Sao Paulo.
PARTICIPANTES: 30 pacientes com diagnostico de oclusao venosa retiniana.
Criterio de Inclusao: Exames de pacientes com diagnostico de oclusao venosa retiniana nos ultimos dois anos.
Criterio de Exclusao: Presenca de outra doenca vascular retiniana concomitante.

PROCEDIMENTOS: Trata-se de revisao de exames de tomografia por coerencia optica de pacientes com oclusao venosa retiniana diagnosticada nos ultimos dois anos no ambulatorio de Retina e Vitreo do Departamento de Oftalmologia e Ciencias Visuais da Universidade Federal de Sao Paulo. Serao analisados mapas angiograficos obtidos a partir de exame de tomografia de coerencia optica (Topcon DRI Triton Optical Coherence Tomography Swept Source) e realizadas medidas do tamanho da zona avascular foveal apresentada em cada caso. As medidas serao feitas a partir de software incluso no proprio equipamento, no qual as imagens estao registradas. Tres examinadores realizarao as medidas de forma independente em dois momentos distintos. Analise

estatistica sera feita para determinar o coeficiente de correlacao intra-classe a fim de determinar o indice de confiabilidade inter-observador e intra-observador das medidas realizadas. (mais informacoes, ver projeto detalhado).

Considerações sobre os Termos de apresentação obrigatória:

1-Foram apresentados adequadamente os principais documentos: folha de rosto; projeto completo; copia do cadastro CEP/UNIFESP, orcamento financeiro.

2-Outros documentos importantes anexados na Plataforma Brasil:

a)Carta da COEP (Outros:<cep1.pdf> POSTAGEM: 29/04/2019).

b)Termo de anonimizacao (Outros: <TERMODEANONIMIZACAO_SIGILO1.docx> Postagem: 29/04/2019)

3– O(A) Pesquisador(a) solicitou a dispensa do TCLE, com a justificativa: “O presente estudo fara uso apenas do banco de dados de exames de tomografia de coerencia optica realizado pelos pacientes com oclusao venosa de retina que fazem acompanhamento nos ambulatorios de Retina e Vitreo, vinculados ao Hospital Sao Paulo e Universidade Federal de Sao Paulo. Nao e possivel obter contato com os pacientes para assinatura de TCLE, pois muitos nao realizam mais seguimento ambulatorial na instituicao. Apresento, dessa forma, termo de anonimizacao e sigilo de dados.”

Conclusões ou Pendências e Lista de Inadequações:

Respostas ao parecer nº 3377370 de 07 de Junho de 2019. PROJETO APROVADO.

PENDÊNCIA 1. No formulário de informações básicas no campo “Benefícios” consta “Não há benefícios envolvidos na participação do presente estudo, pois não há intervenção clínica direta.” Não existe pesquisa que não traga benefício, mesmo que seja de forma indireta. Portanto, é necessário informar os benefícios da pesquisa, mesmo que sejam indiretos (exemplo: “Mesmo não tendo benefícios diretos para o participante, indiretamente está pesquisa contribuirá para a compreensão dos fatores que....”). Por favor, alterar o formulário de informações básicas e incluir a informação sobre os benefícios no projeto (Norma Operacional CNS nº 001 de 2013, item 3.4, 12).

RESPOSTA 1: Corrigida informação quanto ao campo de benefícios. Incluído: “Não há benefícios diretos envolvidos na participação do presente estudo, contudo há benefícios indiretos de contribuição para melhor entendimento das alterações vasculares apresentadas em oclusões venosas retinianas em exame de tomografia de coerência óptica.”

>>> PARECER DO CEP: PENDÊNCIA RESOLVIDA <<<

PENDÊNCIA 2. No projeto consta “Pesquisadores: Bruno Mauricio Rodrigues de Oliveira; Bruno Rebello de Godoy; Alexandre Gomes Bortolotti de Azevedo; Orientadores: Somaia Mitne; Flavio Hirai; Nilva Simeren Bueno de Moraes”. Todavia, no formulário de informações básicas somente consta o nome de Bruno Mauricio Rodrigues de Oliveira. Por favor, incluir o nome de todos no formulário de informações básicas como equipe de pesquisa.

RESPOSTA 2: Corrigida informação quanto aos pesquisadores envolvidos tanto no formulário básico quanto no anexo de projeto detalhado. Equipe de pesquisa: Bruno Mauricio Rodrigues de Oliveira, Bruno Rebello de Godoy, Flavio Hirai e Nilva Simeren Bueno de Moraes.

>>> PARECER DO CEP: PENDÊNCIA RESOLVIDA <<<

PENDÊNCIA 3. O cronograma informado no formulário de informações básicas indica que parte do estudo já será iniciada antes da aprovação do protocolo (Análise dos Exames De OCT - início 22/04/2019). Favor corrigir o formulário. Lembramos que nenhum estudo pode ser iniciado antes da aprovação pelo CEP/UNIFESP (Norma Operacional CNS nº 001 de 2013, item 3.3.f).

RESPOSTA 3: Corrigida informação quanto ao cronograma previsto do estudo tanto no formulário básico quanto no anexo do projeto detalhado. Adicionado que desenho de estudo e análise do banco de dados do OCT: Agosto/2019. Estatística e redação do artigo: Setembro/2019.

>>> PARECER DO CEP: PENDÊNCIA RESOLVIDA <<<

Considerações Finais a critério do CEP:

O CEP informa que a partir desta data de aprovação, é necessário o envio de relatórios parciais (semestralmente), e o relatório final, quando do término do estudo, por meio de notificação pela Plataforma Brasil.

Este parecer foi elaborado baseado nos documentos abaixo relacionados:

Tipo Documento	Arquivo	Postagem	Autor	Situação
Informações Básicas do Projeto	PB_INFORMAÇÕES_BÁSICAS_DO_PROJETO_1320790.pdf	16/06/2019 12:59:16		Aceito
Outros	RESPOSTASPARECERCEP.docx	16/06/2019 12:58:46	Bruno Mauricio Rodrigues de Oliveira	Aceito
Projeto Detalhado / Brochura Investigador	projeto_detalhado.docx	16/06/2019 12:54:27	Bruno Mauricio Rodrigues de Oliveira	Aceito
Outros	TERMODEANONIMIZACAO_SIGILO1.docx	29/04/2019 12:15:37	Bruno Mauricio Rodrigues de Oliveira	Aceito
TCLE / Termos de Assentimento / Justificativa de Ausência	dispensadetcle1.docx	29/04/2019 11:57:27	Bruno Mauricio Rodrigues de Oliveira	Aceito
Outros	cep2.pdf	29/04/2019 11:48:29	Bruno Mauricio Rodrigues de Oliveira	Aceito
Outros	cep1.pdf	29/04/2019 11:48:14	Bruno Mauricio Rodrigues de Oliveira	Aceito
Folha de Rosto	cep3.pdf	29/04/2019 11:47:54	Bruno Mauricio Rodrigues de Oliveira	Aceito

Situação do Parecer:
Aprovado

Necessita Apreciação da CONEP:
Não

SAO PAULO, 09 de Julho de 2019

Assinado por:
Miguel Roberto Jorge
(Coordenador(a))

Endereço: Rua Francisco de Castro, 55
Bairro: VILA CLEMENTINO **CEP:** 04.020-050
UF: SP **Município:** SAO PAULO
Telefone: (11)5571-1062 **Fax:** (11)5539-7162 **E-mail:** cep@unifesp.edu.br
